# Genus- and host-associated codon usage bias patterns in coronavirus spike genes

**DOI:** 10.1371/journal.pone.0351693

**Published:** 2026-06-24

**Authors:** Jia Jun Chew, Chong Han Ng

**Affiliations:** Faculty of Information Science and Technology, Multimedia University, Bukit Beruang, Melaka, Malaysia; Cairo University Faculty of Veterinary Medicine, EGYPT

## Abstract

Codon usage bias (CUB) reflects the combined effects of mutational pressure and natural selection and provides insight into viral evolution and host adaptation. Although previous studies have examined CUB in individual coronaviruses or at the whole-genome level, systematic comparative analyses focusing on the spike (S) gene—an important determinant of viral evolution and host adaptation—across all four coronavirus genera including *Alphacoronavirus, Betacoronavirus, Gammacoronavirus,* and *Deltacoronavirus*, remain limited. In this study, we analyzed CUB in coronavirus spike genes across multiple genera and host groups. Codon usage indices, including codon adaptation index (CAI), effective number of codons (ENC), and GC content at the third synonymous codon position (GC3s), were evaluated alongside multivariate and clustering approaches, including correspondence analysis, hierarchical clustering, heatmap visualization, and ENC–GC3s analysis. Significant differences in CAI and ENC were observed among coronavirus genera, whereas GC3s showed no significant variation, indicating that codon usage patterns are structured primarily by phylogenetic relationships rather than nucleotide composition alone. Multivariate and clustering analyses further supported genus-level organization of codon usage profiles. In contrast, host-based comparisons showed that CAI varied significantly across host groups, while ENC and GC3s remained relatively stable, suggesting that host-associated translational selection influences codon preference without substantially altering overall codon bias strength. Heatmap analysis revealed enrichment of A/U-ending codons and underrepresentation of C/G-ending codons across coronavirus genomes, with consistent suppression of (cytosine-guanine dinucleotides) CpG-containing codons. ENC–GC3s analysis indicated that most genomes deviate from the expected neutral curve, suggesting that factors beyond mutational bias contribute to codon usage patterns. These findings indicate that codon usage bias in coronavirus spike genes is shaped by a combination of virus-intrinsic constraints and host-associated selective pressures, providing a gene-centric, cross-genera framework for understanding coronavirus evolution and host adaptation.

## Introduction

Coronaviruses (family *Coronaviridae*, order *Nidovirales*) are enveloped, positive-sense single-stranded RNA viruses that infect a broad range of mammals and birds. Their genomes, ranging from approximately 27–32 kilobases, are among the largest of all RNA viruses and encode four major structural proteins: spike (S), envelope (E), membrane (M), and nucleocapsid (N), along with several non-structural and accessory proteins. Based on genomic and phylogenetic characteristics, coronaviruses are classified into four genera: *Alphacoronavirus, Betacoronavirus, Gammacoronavirus,* and *Deltacoronavirus*. Among these, *Alpha*- and *Betacoronaviruses* primarily infect mammals, whereas *Gamma*- and *Deltacoronaviruses* are predominantly associated with avian hosts [1,2]. Several members of this family have crossed species barriers to infect humans, including severe acute respiratory syndrome-related coronaviruses (SARS-CoV and SARS-CoV-2) and Middle East respiratory syndrome-related coronavirus (MERS-CoV), causing large-scale outbreaks and ongoing zoonotic threats [3].

The spike (S) protein plays a pivotal role in coronavirus evolution and host adaptation. It is a trimeric class I fusion glycoprotein responsible for mediating viral attachment and entry into host cells through receptor recognition and membrane fusion. Due to its surface exposure and direct interaction with host receptors, the S protein is under strong selective pressure for both immune evasion and receptor adaptation [4,5]. Structural and sequence variability in the S protein drive differences in receptor usage, such as angiotensin-converting enzyme 2 (ACE2) and dipeptidyl peptidase 4 (DPP4), which underlie host specificity and cross-species transmission [6–8]. As viruses rely entirely on host translational machinery, evolutionary changes in the spike gene, including synonymous codon usage, can influence translation efficiency, viral fitness, and the ability to adapt to new hosts [9]. Consequently, codon usage analysis of the S gene provides key insights into coronavirus evolution and host adaptation.

One important molecular signature of host specificity is codon usage bias (CUB), defined as the non-random usage of synonymous codons in protein-coding sequences. This phenomenon is widespread across organisms, including viruses, and reflects underlying evolutionary pressures acting on genomes [9–11]. CUB arises from a combination of mutational bias, reflecting nucleotide composition constraints, and natural selection for translational efficiency, accuracy, and compatibility with host tRNA pools [12]. In viruses, codon usage patterns are strongly shaped by adaptation to host cellular environments, as efficient replication depends on compatibility between viral codon preferences and host translational systems. In RNA viruses, CUB can influence replication efficiency, immune recognition, and host range [9].

However, viral codon usage is not shaped solely by translational selection. It is also influenced by mutational pressure, genomic GC content, and innate immune constraints. For example, RNA viruses such as HIV-1 suppress CpG dinucleotides because these motifs can be recognized by host antiviral systems, including zinc-finger antiviral protein (ZAP), which selectively targets CpG-rich viral RNA for degradation [13]. As a result, synonymous codon choice often reflects a balance between maximizing translation efficiency and minimizing immune detection.

Synonymous codon usage can also affect viral phenotype beyond protein abundance. Rare codons may slow ribosome movement, influencing co-translational folding, glycosylation, and structural maturation of proteins. Consequently, synonymous mutations may alter infectivity or antigenicity without changing the amino acid sequence [14].

Recent studies have shown that coronaviruses, including SARS-CoV-2, tend to prefer A/U-ending codons and exhibit relatively low codon adaptation to human hosts, suggesting incomplete translational optimization following zoonotic spillover [15,16]. Large-scale analyses have also demonstrated that the S gene exhibits distinct codon usage signatures and elevated nonsynonymous substitution rates relative to other viral genes [17,18], consistent with stronger selective pressures related to receptor binding and immune interactions.

While numerous studies have examined codon usage bias in coronaviruses—ranging from genome-wide analyses of individual species such as SARS-CoV-2 to investigations of structural genes—systematic, cross-genera studies focusing specifically on the spike (S) protein remain limited. For example, previous work has characterized codon usage patterns in SARS-CoV-2 and related viruses, showing relatively low codon usage bias shaped by both mutational pressure and natural selection within *Betacoronavirus* lineages [19]. One comprehensive analysis did investigate codon usage across a broad sample of *Orthocoronavirinae* genomes spanning all four genera (*Alpha*-, *Beta*-, *Gamma*-, and *Deltacoronavirus*). However, this study focused on general codon usage patterns in whole genomes, rather than integrating codon bias metrics specifically for the S gene with phylogenetic context [20].

In contrast, this study systematically integrates codon usage bias analysis of the spike (S) gene with phylogenetic comparisons across all four coronavirus genera (*Alphacoronavirus, Betacoronavirus, Gammacoronavirus*, and *Deltacoronavirus*). This genus-wide, gene-centric approach provides a unified evolutionary perspective on how synonymous codon usage in the spike protein relates to phylogenetic divergence and host adaptation. The high sequence variability of the S gene, combined with its essential role in host interaction, makes it a suitable model for investigating how molecular evolution shapes viral adaptation [21]. Comparative analysis of the S gene may reveal whether codon usage variation corresponds to evolutionary divergence among coronavirus genera and how selection acts on translational features to facilitate host switching or antigenic evolution.

In this study, we calculated codon usage indices of the spike genes from representative coronavirus genomes across the four genera, including ENC, GC3s, RSCU, and CAI. By comparing these indices across genera and host groups, we aim to elucidate the relative contributions of mutational bias and natural selection in shaping codon usage patterns. Understanding these evolutionary dynamics will contribute to a better understanding of coronavirus host adaptation, host-range expansion, and the potential emergence of novel zoonotic strains.

## Materials and methods

### Data acquisition and sequence retrieval

Representative coronavirus genomes were obtained from the NCBI Virus database (https://www.ncbi.nlm.nih.gov/labs/virus/vssi/) to encompass the taxonomic diversity of the four recognized genera within the subfamily *Orthocoronavirinae*: *Alphacoronavirus, Betacoronavirus, Gammacoronavirus*, and *Deltacoronavirus*. Genome records were retrieved on 2^nd^ April 2025 using the query: (“*Orthocoronavirinae*”[Organism]) AND (“complete genome”[Properties] OR “reference genome”[Properties]). To minimize redundancy and overrepresentation of closely related taxa, one representative genome was selected per coronavirus species. A total of 74 complete genomes were initially retrieved. Annotated coding sequences (CDS) were downloaded in GenBank/RefSeq format, and spike (S) gene CDS regions were extracted using Biopython v1.81 based on feature annotations [22]. Extracted spike gene sequences were subjected to quality-control filtering. Sequences were excluded if they contained incomplete CDS annotations, internal stop codons, or ambiguous nucleotides exceeding 1% of sequence length. After filtering, 64 high-quality spike gene sequences were retained for downstream analyses (S1 Table).

### Computation of codon usage bias metrics

Codon usage indices including relative synonymous codon usage (RSCU), effective number of codons (ENC), and GC content at the third synonymous codon position (GC3s) were calculated using CodonW v1.4.2. Codon adaptation index (CAI) values were estimated using the CAIcal server with codon usage tables obtained from the Kazusa Codon Usage Database [23]. CAI estimates the similarity of codon usage to a reference set of highly expressed host genes. High CAI value indicates strong adaptation to host codon preference while low CAI value may be due to reservoir viruses, broad host range or less optimized translation. ENC ranges from 20 to 61, with lower values indicating stronger codon usage bias. GC3s reflects nucleotide composition at synonymous sites, with higher values indicating GC-rich mutational bias or host adaptation and lower values indicating AT-rich tendencies.

To assess host-specific translational adaptation, CAI was calculated using host-specific codon usage tables as reference sets. For each viral sequence, the reference corresponded to its most probable natural host based on reported primary host information from NCBI metadata and published literature (e.g., *Homo sapiens* for human coronaviruses, *Sus scrofa* for porcine coronaviruses, *Bos taurus* for bovine coronaviruses, *Felis catus* for feline coronaviruses). For host groups lacking species-specific codon usage data, a representative species was used (e.g., *Rhinolophus ferrumequinum* for bat-associated viruses and *Mus musculus* for rodent-associated viruses). The full list of reference sets is provided in S2 Table. Because several host species were represented by small sample sizes, hosts were consolidated into broader biologically meaningful groups to improve statistical power: bat (n = 25), avian (n = 13), human (n = 7), rodent/insectivore (rodent: n = 7; shrew: n = 2; hedgehog: n = 1; total n = 10), and other mammals (pig: n = 4; cat: n = 1; rabbit: n = 1; whale: n = 1; mustelids: n = 1; bovine: n = 1; total n = 9). All outputs were compiled into a single dataset for statistical analyses in R v4.3.1.

### Statistical analysis

Differences in ENC, CAI, and GC3s across genera and host groups were assessed using the non-parametric Kruskal–Wallis test, which is robust to non-normal distributions and unequal sample sizes. When significant, Dunn’s post hoc tests with Bonferroni correction were applied to identify pairwise differences. Statistical analyses were performed in R (v4.3.1) using tidyverse for data handling, ggpubr for statistical annotation, and ggthemes for visualization. Results were considered significant at p < 0.05.

### Correspondence analysis

Correspondence analysis (CA) was performed on RSCU values to investigate multivariate patterns of synonymous codon usage [24]. Non-informative codons (AUG, UGG, and stop codons) were excluded, resulting in 59 synonymous codons across 64 genomes. CA was conducted using the FactoMineR package in R v4.3.1. The first two dimensions, explaining the largest proportion of variance (inertia), were retained for visualization. Genomes were projected into reduced multivariate space and colored according to coronavirus genus or host category.

### Hierarchical clustering analysis

Hierarchical clustering of RSCU profiles was performed using the pvclust package in R v4.3.1 [25]. Pairwise dissimilarities were calculated using Euclidean distance, and clusters were constructed using Ward’s minimum variance method (Ward.D2). Cluster stability was assessed using multiscale bootstrap resampling (n = 1000). Both bootstrap probability (BP) values and approximately unbiased (AU) *p*-values were calculated. The AU *p*-value is derived from multiscale bootstrap resampling and provides a less biased estimate of cluster support compared to standard bootstrap values. It adjusts for sample size and model variability, making it a more reliable measure of cluster robustness. Clusters with AU ≥ 95% were considered strongly supported. Dendrograms were visualized using ggtree and annotated by coronavirus genus and host origin.

### Heatmap analysis

Codon usage variation was further examined using heatmap visualization of RSCU values with the pheatmap package in R v4.3.1 [26]. Rows (genomes) were clustered using Euclidean distance and Ward.D2 linkage. Codons were labeled in the format “Codon (amino acid)”. A three-color gradient centered at RSCU = 1 was used, where values >1 indicate preferred codons and values <1 indicate underrepresented codons.

### ENC–GC3s analysis

To evaluate the relative contributions of mutational pressure and natural selection, ENC values were plotted against GC3s. The expected ENC under neutrality was calculated according to the formula from Wright [27]:


$$\begin{array}{c} ENC_{expected}=\text{2}+GC\text{3}s+\frac{\text{2}\text{9}}{GC\text{3}s^{\text{2}}+(\text{1}-GC\text{3}s{)}^{\text{2}}} \end{array}$$

A ± 2 ENC-unit interval around the theoretical curve was used as a graphical heuristic to indicate sequences approximating neutrality. Genomes lying near the curve were interpreted as primarily influenced by nucleotide compositional constraints, whereas those substantially below the curve were considered to be influenced by additional factors, including natural selection, dinucleotide bias, and lineage-specific evolutionary pressures. Scatterplots were generated using ggplot2 in R, with genomes colored by genus and shaped according to host category.

## Results

### Comparison of codon usage bias across genera

**Fig 1:**
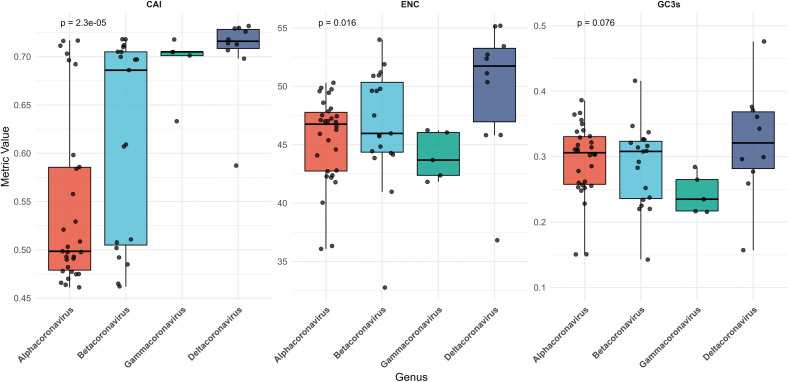
Codon usage bias of spike (S) genes across coronavirus genera. Boxplots show CAI, ENC, and GC3s distributions among *Alphacoronaviruses, Betacoronaviruses, Gammacoronaviruses,* and *Deltacoronaviruses*. Differences were assessed using the Kruskal–Wallis test with Dunn’s post hoc comparisons (Bonferroni-corrected). CAI *(p* < 0.0001) and ENC (*p* < 0.05) differed significantly among genera, whereas GC3s did not (*p* > 0.05). Boxes indicate IQR, center lines show medians, and whiskers extend to 1.5 × IQR.

Codon usage bias (CUB) of coronavirus spike (S) genes was assessed across genera using codon adaptation index (CAI), effective number of codons (ENC), and GC content at the third synonymous codon position (GC3s) (Fig 1, S3 Table). CAI values were interpreted as comparative indicators of host translational compatibility rather than definitive measures of adaptation. CAI values ranged from 0.46 to 0.73, suggesting variability in the degree of host-related codon adaptation among coronaviruses. ENC values ranged from 32.77 to 55.19, consistent with weak to moderate codon usage bias across genera. GC3s values ranged from 14.3% to 47.6%, reflecting heterogeneity in nucleotide composition among coronavirus lineages.

To evaluate inter-genus differences, a Kruskal–Wallis test was performed, followed by Dunn’s post hoc test with Bonferroni correction (Table 1; S4 Table). Significant differences were observed among genera for both CAI and ENC (*p* < 0.05), whereas GC3s did not differ significantly (*p* > 0.05) (Fig 1). Post hoc analysis revealed that *Deltacoronaviruses* exhibited significantly higher ENC values than both *Alphacoronaviruses* (*p* = 0.038) and *Gammacoronaviruses* (*p* = 0.035), indicating comparatively weaker codon usage bias in *Deltacoronaviruses* (Table 1). In addition, CAI differed significantly between *Alphacoronaviruses* and *Deltacoronaviruses* (*p* < 0.001), as well as between *Betacoronaviruses* and *Deltacoronaviruses* (*p* = 0.035), suggesting variation in host-related codon adaptation across genera. No significant pairwise differences were observed for GC3s, indicating relatively conserved nucleotide composition at synonymous sites.

**Table 1:** Dunn’s post-hoc pairwise comparisons of ENC and CAI values among coronavirus genera (Bonferroni adjusted).

Comparison	Adjusted *p* value (ENC)	Adjusted *p* value (CAI)
***Alphacoronavirus* vs. *Deltacoronavirus***	0.038	0.001
***Gammacoronavirus* vs. *Deltacoronavirus***	0.035	ns
***Betacoronavirus* vs. *Deltacoronavirus***	ns	0.035
**All other pairwise comparisons**	ns	

Note: *p*-values adjusted via Dunn’s post hoc test with Bonferroni correction. ns = not significant (*p* > 0.05).

### Comparison of codon usage bias across host groups

**Fig 2:**
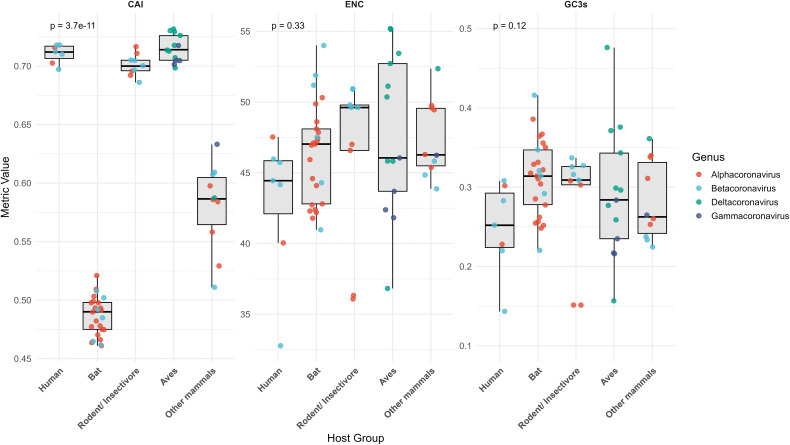
Codon usage bias of spike (S) genes across coronavirus host groups. Boxplots show CAI, ENC, and GC3s distributions among human, bat, rodent/insectivore (rodent, shrew, hedgehog), avian, and other mammals (pig, cat, rabbit, whale, mustelids, bovine) groups. Differences were assessed using the Kruskal–Wallis test with Dunn’s post hoc comparisons (Bonferroni-corrected). CAI differed significantly (*p* < 3.7 × 10^−11^), whereas ENC and GC3s did not (*p* > 0.05). Post hoc analysis indicated lower CAI values in bat-associated coronaviruses. Boxes indicate IQR, center lines show medians, and whiskers extend to 1.5 ×  IQR.

To assess host-associated variation in codon usage bias, coronavirus spike (S) gene sequences were grouped into five biologically relevant host categories based on their primary natural host: human, bat, rodent/insectivore (rodent, shrew, hedgehog), avian, and other mammals (pig, cat, rabbit, whale, mustelids, bovine) (Fig 2; S5 Table). Codon usage indices (CAI, ENC, and GC3s) were compared among groups using the Kruskal–Wallis test.

A highly significant difference was observed for CAI (*p* < 3.7 × 10^−11^), indicating substantial host-associated variation in codon adaptation. In contrast, ENC (*p* = 0.33) and GC3s (*p* = 0.12) showed no significant differences across host groups, suggesting that the overall magnitude of codon usage bias and nucleotide composition at synonymous sites are relatively conserved among hosts. Post hoc Dunn’s tests with Bonferroni correction showed that bat-associated coronaviruses exhibited significantly lower CAI values than other host groups, including human and avian viruses (Table 2; S6 Table). In contrast, human- and avian-associated viruses showed higher CAI values, while rodent/insectivore-associated viruses displayed intermediate values. Viruses from other mammals exhibited moderate CAI levels. No significant pairwise differences were detected for GC3s or ENC after multiple testing correction.

**Table 2:** Dunn’s post-hoc pairwise comparisons of CAI values among coronavirus host groups (Bonferroni adjusted).

Comparison	Adjusted *p* value
**Human vs. Bat**	< 0.001
**Bat vs. Rodent/ Insectivore**	< 0.001
**Bat vs. Aves**	< 0.001
**Aves vs. Other mammals**	0.0275
**All other pairs**	>0.05

Note: p-values adjusted via Dunn’s post hoc test with Bonferroni correction. ns = not significant (*p* > 0.05).

### Correspondence analysis of codon usage patterns

**Fig 3:**
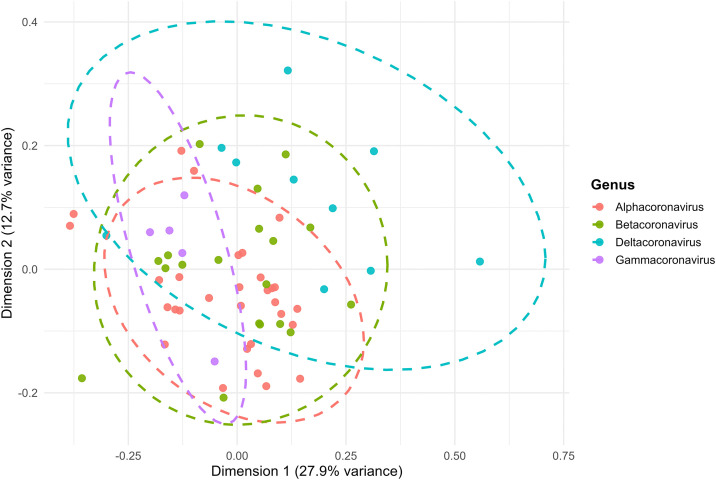
Correspondence analysis (CA) of relative synonymous codon usage (RSCU) values across coronavirus genera. Points represent viral genomes coloured by genus. The first two CA dimensions explain 40.6% of the total variation (Dimension 1: 27.9%; Dimension 2: 12.7%). *Alphacoronaviruses* and *Betacoronaviruses* show partial overlap and broader dispersion, whereas *Gammacoronaviruses* and *Deltacoronaviruses* form more distinct clusters with varying degrees of spread, indicating differences in codon usage patterns among genera.

Correspondence analysis (CA) of RSCU values revealed structured variation in codon usage among coronavirus genera (Fig 3). The first two dimensions explained 40.6% of the total inertia (Dimension 1: 27.9%; Dimension 2: 12.7%). *Alphacoronaviruses* and *Betacoronaviruses* formed partially overlapping clusters near the origin, indicating similar codon usage profiles. In contrast, *Deltacoronaviruses* were distributed along the positive axis of Dimension 1 and showed broader dispersion. *Gammacoronaviruses* formed a more compact cluster, separated primarily along Dimension 2. Overall, dispersion patterns differed among genera, with *Deltacoronaviruses* showing the widest spread and *Gammacoronaviruses* forming the most compact cluster.

Dimension 1 reflected an AU-to-GC synonymous codon gradient, with AU-ending codons (UUA, −0.240; AGA, −0.217; AGU, −0.216) at the negative pole and C/G-ending codons (CGC, + 0.426; CUC, + 0.418; UCC, + 0.365; CGG, + 0.363) at the positive pole. Dimension 2 contrasted A-ending codons (GGA, + 0.362; AGA, + 0.288; CUA, + 0.285) against CpG-containing codons (GCG, −0.206; CGU, −0.194; ACG, −0.162), with four of five *Gammacoronaviruses* clustering at the positive pole; the single exception, beluga whale coronavirus SW1, occupied the negative pole consistent with its mammalian host.

### Hierarchical clustering of RSCU profiles

**Fig 4:**
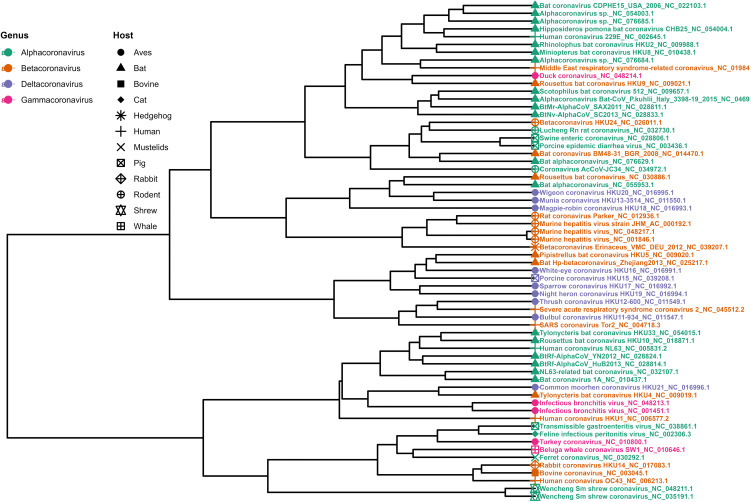
Hierarchical clustering of coronavirus spike (S) genes based on relative synonymous codon usage (RSCU) profiles. The dendrogram was constructed using Euclidean distance and Ward.D2 linkage, with cluster support assessed by multiscale bootstrap resampling (1,000 replicates) using the pvclust algorithm. Clusters with approximately unbiased (AU) p ≥ 95% are indicated. Tip colours represent viral genera, and distinct host species (avian, bat, bovine, cat, hedgehog, human, mustelid, pig, rabbit, rodent, shrew, and whale) are denoted by different symbols. The dendrogram shows genus-level grouping with additional subclustering within genera.

To further examine codon usage patterns, hierarchical clustering was performed on the RSCU profiles of coronavirus spike (S) genes using Euclidean distance and Ward.D2 linkage (Fig 4). Cluster robustness was evaluated using multiscale bootstrap resampling (n = 1000), with several internal nodes showing strong support (approximately unbiased (AU) p ≥ 95%). The resulting dendrogram revealed clear genus-level organization of codon usage patterns. *Deltacoronaviruses* formed a well-supported cluster, while *Gammacoronaviruses* also grouped into a distinct clade. Within *Deltacoronaviruses*, porcine *deltacoronavirus* clustered with avian-associated viruses (e.g., white-eye, sparrow, thrush, and night-heron coronaviruses). Similarly, within *Gammacoronaviruses*, beluga whale coronavirus SW1 clustered with avian infectious bronchitis virus (IBV). *Betacoronaviruses* were further subdivided into recognizable subclades. Murine hepatitis viruses grouped together, while human coronavirus OC43 clustered with bovine coronavirus. SARS-related *betacoronaviruses* formed a separate cluster with bat SARS-related viruses, and MERS-related viruses grouped with bat HKU4 and HKU5 viruses. In contrast, *Alphacoronaviruses* showed greater dispersion, forming multiple clusters. Human coronaviruses (HCoV-229E and HCoV-NL63) clustered with bat-associated *alphacoronaviruses*, while a separate cluster included feline, canine, and transmissible gastroenteritis virus sequences. Bat-associated *alphacoronaviruses* were further partitioned into several subclusters, indicating substantial intra-genus variation.

### Heatmap analysis of codon usage bias

**Fig 5:**
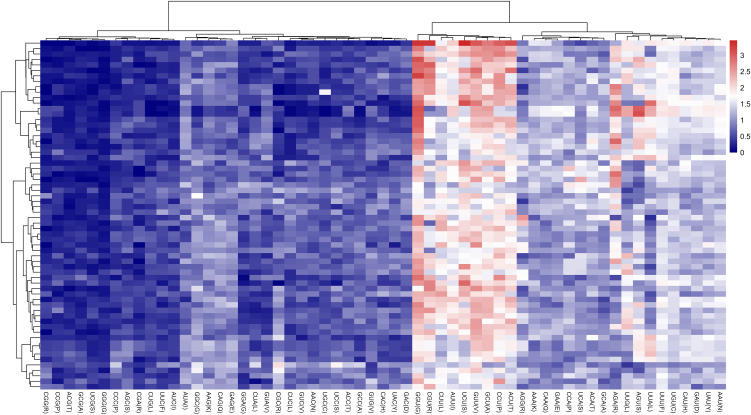
Heatmap of RSCU values across coronavirus spike (S) genes. Rows represent viral genomes grouped by genus, and columns correspond to codons. Colours indicate codon usage bias (RSCU > 1, overrepresented; RSCU < 1, underrepresented). Hierarchical clustering of rows and columns reveals patterns of codon usage across genera, including a general enrichment of A/U-ending codons.

To further characterize codon usage patterns, a heatmap of relative synonymous codon usage (RSCU) values was generated for coronavirus spike (S) genes (Fig 5). Hierarchical clustering of both codons and viral genomes revealed structured patterns of synonymous codon usage across genera. The heatmap showed a clear global pattern of codon preference, with a subset of codons consistently overrepresented (RSCU > 1) and others underrepresented (RSCU < 1) across coronavirus genomes. In particular, A/U-ending codons were generally enriched, whereas C/G-ending codons were underrepresented. This bias was evident across multiple amino acids. For example, leucine was preferentially encoded by UUA and CUU, while CUA, CUC, CUG, and UUG were less frequently used. Similarly, arginine showed higher usage of AGA and CGU, with reduced usage of AGG, CGC, CGA, and CGG. Comparable trends were observed for other amino acids, including isoleucine (AUU), serine (UCU), threonine (ACU), proline (CCU), alanine (GCU), valine (GUU), and glycine (GGU), where A/U-ending codons were more frequently used. In addition, codons containing CpG dinucleotides (e.g., CGG, CCG, ACG) were consistently underrepresented across coronavirus genomes (Table 3). For amino acids encoded by only two synonymous codons, such as asparagine, aspartate, histidine, tyrosine, cysteine, glutamine, phenylalanine, and lysine, neither codon consistently exceeded RSCU = 1 across all genera, reflecting near-equal usage without strong directional preference. Clustering of RSCU profiles further revealed grouping patterns among viral sequences, consistent with differences in codon usage among genera.

**Table 3:** Summary of preferred and less-preferred codons in coronavirus S genes.

Amino Acid	Preferred Codon(s) (RSCU > 1, Red)	Less Preferred Codon(s) (RSCU < 1, Blue)
**Leu (L)**	UUA, CUU	CUA, CUC, CUG, UUG
**Arg (R)**	AGA, CGU	AGG, CGC, CGA, CGG
**Ile (I)**	AUU	AUA, AUC
**Ser (S)**	UCU	UCA, UCC, UCG, AGC, AGU
**Thr (T)**	ACU	ACA, ACC, ACG
**Pro (P)**	CCU	CCA, CCC, CCG
**Ala (A)**	GCU	GCA, GCC, GCG
**Val (V)**	GUU	GUA, GUC, GUG
**Gly (G)**	GGU	GGA, GGG, GGC
**Asn (N)**	–	AAC, AAU
**Asp (D)**	–	GAC, GAU
**Glu (E)**	–	GAA, GAG
**His (H)**	–	CAC, CAU
**Tyr (Y)**	–	UAC, UAU
**Cys (C)**	–	UGC, UGU
**Gln (Q)**	–	CAA, CAG
**Phe (F)**	–	UUC, UUU
**Lys (K)**	–	AAA, AAG

### ENC–GC3s analysis of codon usage bias

**Fig 6:**
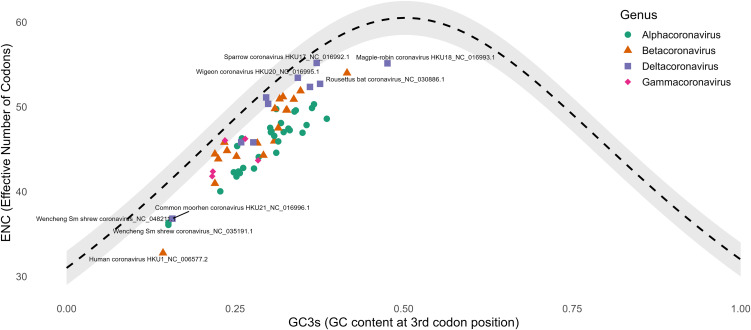
ENC–GC3s analysis of coronavirus genomes. The dashed line represents the expected ENC curve under neutral mutation pressure. The shaded region indicates a ± 2 ENC-unit neutrality zone around the theoretical curve. Each point represents a coronavirus genome, colored and shaped according to genus. Most genomes lie below the expected curve, suggesting that factors beyond mutational pressure, including natural selection, contribute to codon usage bias in coronavirus spike genes.

ENC–GC3s analysis was performed to assess the relative contributions of mutational bias and selection (Fig 6). ENC values ranged from 32.77 to 55.19, indicating weak to moderate codon usage bias. None of the sequences approached the maximum ENC value of 61 expected under no bias. Most genomes were distributed below the expected ENC curve, suggesting that codon usage patterns are not explained solely by mutational bias. Genus-level differences were observed. *Alphacoronaviruses* and *Betacoronaviruses* showed broader dispersion, with several genomes positioned well below the curve. In contrast, *Deltacoronaviruses* and *Gammacoronaviruses* tended to cluster closer to the expected curve, with generally higher ENC values. At the individual genome level, some viruses (e.g., human coronavirus HKU1 and Wencheng shrew coronavirus) showed pronounced deviation below the curve, whereas several avian *deltacoronaviruses* were located closer to the expected relationship.

## Discussion

### Determinants of codon usage bias in coronavirus spike genes

This study demonstrates that codon usage bias (CUB) in coronavirus spike (S) genes is structured by a hierarchical interplay between phylogenetic constraints and host-associated selection. Across all analytical frameworks—including codon usage indices (Figs 1 and 2), correspondence analysis (Fig 3), hierarchical clustering (Fig 4), heatmap patterns (Fig 5), and ENC–GC3s relationships (Fig 6)—a consistent pattern emerges in which phylogeny establishes the baseline codon usage landscape, while host-related factors fine-tune codon preference without fundamentally altering overall bias strength. Unlike previous studies that focused primarily on SARS-CoV-2 or whole-genome analyses within individual coronavirus lineages, or cross-genera analyses that did not integrate gene-specific and host-level comparisons [20,28,29], the present study integrates multiple codon usage approaches across all four coronavirus genera using the spike gene as a common evolutionary framework. This comparative design provides broader insight into how synonymous codon usage evolves in relation to both viral ancestry and host adaptation.

At the genus level, significant differences in CAI and ENC, but not GC3s, indicate that codon usage patterns are lineage-specific and evolutionarily conserved, rather than primarily driven by nucleotide composition alone. The strong concordance between correspondence analysis and hierarchical clustering reinforces this interpretation, as viral sequences consistently group according to genus. This phylogenetic structuring of codon usage has been widely reported in RNA viruses, where lineage-specific constraints often dominate over host effects [12]. Comparable patterns have also been observed in SARS-CoV-2 and related *betacoronaviruses*, where codon usage signatures closely reflect phylogenetic relationships despite circulation in different hosts [29].

The codon-level drivers of these patterns are further revealed by correspondence analysis. Dimension 1 reflects the AU-to-GC mutational bias gradient characteristic of coronavirus evolution, while Dimension 2 captures a distinct A-ending codon preference in avian *Gammacoronaviruses*, consistent with host-specific translational selection acting through differences in tRNA availability between avian and mammalian hosts [30–32]. The positioning of beluga whale coronavirus SW1 at the opposite Dim2 pole reinforces that host environment shapes codon preference independently of genus-level phylogeny. The partial overlap between *Alphacoronaviruses* and *Betacoronaviruses,* contrasted with the compact cluster of *Gammacoronaviruses* and the broad dispersion of *Deltacoronaviruses*, further suggests that evolutionary divergence among genera is reflected in their synonymous codon preferences. Together, these multivariate patterns confirm that synonymous codon usage in the spike gene carries a strong phylogenetic signal that persists across host boundaries.

In contrast, host-based comparisons reveal that CAI varies significantly across host groups, whereas ENC and GC3s remain relatively stable. This indicates that host-associated selection primarily influences codon preference rather than the overall magnitude of codon bias or nucleotide composition. Such a pattern is consistent with translational selection acting through host tRNA availability and tRNA modification dynamics, where synonymous codon usage is optimized to enhance translational efficiency without reshaping genome-wide compositional constraints [9,10,31,32]. The lower CAI values observed in bat-associated coronaviruses likely reflect reduced translational specialization in reservoir hosts, where maintaining broader host compatibility may be advantageous. Since 19 of the 25 bat-associated coronaviruses in this dataset which are the largest host group, belongs to *Alphacoronavirus* (S1 Table), the comparatively lower CAI values observed in bat-associated viruses may partly reflect genus-level codon usage constraints characteristic of *Alphacoronavirus* rather than host-specific translational adaptation alone.

Conversely, higher CAI values in human- and avian-associated viruses suggest stronger adaptation to host-specific translational environments, potentially reflecting prolonged circulation and evolutionary fine-tuning within those hosts. These findings are biologically important because they suggest that synonymous codon usage may contribute to host range flexibility and the capacity for cross-species transmission.

The heatmap analysis reveals a pervasive enrichment of A/U-ending codons and suppression of C/G-ending codons across coronavirus genomes, consistent with the AU-rich composition characteristic of many RNA viruses [12]. Similar A/U-ending codon preferences have been widely reported in SARS-CoV-2 and other coronaviruses [29,33], suggesting that AU-richness represents a conserved compositional feature of coronavirus evolution rather than a lineage-specific phenomenon.

In addition, the marked underrepresentation of CpG-containing codons supports a role for host immune selection. CpG motifs are recognized by host antiviral mechanisms such as zinc-finger antiviral protein (ZAP), which targets CpG-rich RNA for degradation [13,34]. The consistent depletion of CpG-containing codons across all four coronavirus genera in the present study therefore suggests that immune-associated selective pressures may act broadly across coronavirus evolution rather than being restricted to recently emerged zoonotic viruses.

The ENC–GC3s analysis provides direct evidence that codon usage bias is not governed solely by mutational pressure. Although some genomes lie near the expected neutrality curve, the majority fall below it, indicating the influence of additional selective forces. Similar deviations from neutrality have been reported in SARS-CoV-2 and other viruses, where both mutational bias and natural selection contribute to codon usage evolution [9,29,35]. At the individual genome level, the particularly pronounced deviations of HCoV-HKU1 and Wencheng shrew coronavirus below the neutral curve warrant specific consideration. The pronounced deviation of HKU1 may reflect its prolonged circulation in human hosts [36], during which sustained translational selection has shaped spike codon usage beyond what its AU-rich nucleotide composition alone would predict [37]. Similarly, the strong deviation of Wencheng shrew coronavirus living in the physiologically distinctive insectivore host environment may impose unique selective pressures on viral codon usage [38].

These findings extend those of Daron & Bravo [20], who analyzed whole-genome codon usage across all four coronavirus genera and concluded that mutational bias and CpG-directed selection were the predominant drivers of codon usage variability. While the present study confirms a role for both processes, the spike-gene-specific ENC–GC3s patterns suggest that translational selection constitutes an additional contributor beyond what whole-genome analyses identified — a discrepancy likely reflecting the distinct and stronger selective pressures acting on the spike protein relative to the broader genome.

At the genus level, *Alphacoronaviruses* and *Betacoronaviruses* exhibit broader dispersion and more pronounced deviations from the expected curve, suggesting stronger and more heterogeneous selection pressures within these genera. In contrast, *Deltacoronaviruses* cluster closer to the theoretical expectation and display higher ENC values, consistent with weaker codon usage bias and a greater contribution of mutational processes. These patterns are congruent with the genus-level differences observed in ENC and the dispersion patterns identified in correspondence analysis.

Notably, the clustering of viruses across host boundaries—for example, porcine *deltacoronavirus* grouping with avian-associated viruses and beluga whale coronavirus clustering with avian infectious bronchitis viruses—indicates that codon usage patterns can remain conserved despite host shifts. This supports the view that evolutionary history constrains codon usage more strongly than immediate host environment, consistent with studies showing that codon usage patterns in RNA viruses are shaped by long-term evolutionary and lineage-specific constraints rather than simple host matching [30]. Such phylogenetic inertia may limit the speed at which viruses fully optimize codon usage following host jumps.

Taken together, these findings support a hierarchical model of codon usage evolution in coronaviruses: mutational bias establishes an AU-rich compositional background, phylogenetic inheritance preserves lineage-specific codon usage patterns, and host-driven selection fine-tunes codon preference to optimize translation and evade host defenses. This framework reconciles the coexistence of strong lineage conservation with detectable host-associated adaptation.

### Limitations

Several limitations should be considered. First, the analysis focuses exclusively on the spike (S) gene, which, while functionally critical, may not fully represent genome-wide codon usage patterns. Different viral genes can be subject to distinct selective pressures, particularly those involved in replication versus host interaction. Second, sampling across genera and host groups is uneven, which may influence statistical power and clustering resolution. In particular, smaller host categories may reduce sensitivity for detecting subtle differences in ENC and GC3s. Third, CAI was used as a proxy for host translational adaptation based on available codon usage tables. However, CAI does not account for tissue-specific expression, dynamic tRNA abundance, or infection-stage variability, and thus provides only an approximate measure of translational compatibility. Finally, the study is based on computational analyses and cannot directly establish causal relationships between codon usage patterns and viral fitness, replication efficiency, or host adaptation. Functional validation is therefore required to confirm the biological significance of the observed patterns.

### Future directions

Future work should extend this analysis to whole genomes to determine whether the hierarchical patterns observed in the spike gene are consistent across other viral genes with different functional roles. Integrating host-specific tRNA abundance profiles and transcriptomic data would provide a more mechanistic understanding of translational selection and allow more precise evaluation of codon adaptation beyond CAI-based inference. Experimental approaches, including reverse genetics and codon deoptimization studies, could directly test how synonymous codon changes influence viral replication, protein folding, and host adaptation. Finally, investigating the interaction between codon usage bias and immune evasion mechanisms—particularly CpG suppression and ZAP-mediated restriction—may provide deeper insight into how codon usage contributes to viral emergence and cross-species transmission.

## Conclusion

This study systematically characterized codon usage bias (CUB) in coronavirus spike (S) genes across genera and host groups using multiple complementary analytical approaches. Codon usage patterns were strongly structured by phylogenetic relationships, with significant differences in CAI and ENC among genera, while GC3s remained relatively conserved. In contrast, host-based comparisons showed significant variation in CAI but not in ENC or GC3s, suggesting that host-associated translational selection influences codon preference without substantially altering overall codon bias or nucleotide composition.

Across all genomes, A/U-ending codons were preferentially used, whereas C/G-ending and CpG-containing codons were underrepresented, consistent with the combined effects of mutational bias and host-related selective pressures. ENC–GC3s analysis further indicated that codon usage patterns cannot be explained by mutational pressure alone, suggesting contributions from additional evolutionary forces.

Overall, these findings demonstrate that codon usage bias in coronavirus spike genes reflects an interplay between phylogenetic constraints and host-associated selection, providing insight into viral evolution and host adaptation.

## Supporting information

S1 Table Metadata and codon usage metrics of coronavirus spike (s) genes. (XLSX)

S2 Table Host specific codon usage reference for codon adaptation index calculations. (XLSX)

S3 Table Summary statistics of codon usage indices across coronavirus genera. (XLSX)

S4 Table Dunn’s post hoc pairwise comparisons of codon usage indices among coronavirus genera. (XLSX)

S5 Table Summary statistics of codon usage indices across coronavirus host groups. (XLSX)

S6 Table Dunn’s post hoc pairwise comparisons of codon usage indices among coronavirus host groups. (XLSX)
