# Supplementary material for: Genus- and host-associated codon usage bias patterns in coronavirus spike genes
Source: PLoS One. 2026 Jun 24;21(6):e0351693. doi: 10.1371/journal.pone.0351693 (PMC13293466; doi:10.1371/journal.pone.0351693)
Supplement: Attachment — Submitted filename: Response to Reviewers.docx [file pone.0351693.s007.docx]

**RESPONSES TO REVIEWERS COMMENTS**

We sincerely thank the reviewers for their careful evaluation and constructive comments. We have revised the manuscript accordingly and believe that these changes have significantly improved its clarity, accuracy, and overall quality. All changes have been incorporated into the revised manuscript and are highlighted where appropriate.

**Reviewer #1**: I have reviewed the manuscript ID PONE-D-26-10054 titled "Genus- and host-associated codon usage bias patterns in coronaviruses spike genes" submitted by Jia Jun Chew and collaborator. The paper describes codon usage bias patterns in coronaviruses spike genes associated to its hosts. Codon usage patterns were assessed of *Alphacoronavirus, Betacoronavirus, Gammacoronavirus*, and *Deltacoronavirus* using effective number of codons (ENC), relative synonymous codon usage (RSCU), codon adaptation index (CAI), correspondence analysis, hierarchical clustering, and GC content at synonymous third codon positions (GC3s) modelling. The authors pointing out the dual role of mutation and natural selection in coronavirus evolution, with host-driven selection as a major determinant of codon bias.

Although the work described has significance, the manuscript is well written. However, in some area needs to be polished as there are grammatical errors in it.

1. What was reason behind confining study for 74 reference genome?

**RE:** We thank the reviewer for this important question. The dataset was not arbitrarily limited to 74 genomes. Instead, all available reference genomes under the query “*Orthocoronavirinae*” were retrieved from the NCBI Virus database to ensure high-quality, well-annotated, and non-redundant sequences across the four coronavirus genera. *Orthocoronavirinae* is the currently accepted subfamily name in modern coronavirus taxonomy. “*Coronavirinae*” is an older classification that was revised by the International Committee on Taxonomy of Viruses.

To avoid overrepresentation of closely related strains, one representative genome per species was selected. After quality filtering (removal of incomplete or low-quality spike CDS), 64 genomes were retained for downstream analyses. This clarification has now been added to the Materials and Methods section.

2. Highlight the significance of synonymous codons ending in A/T & C/G in the discussion and

provide similarity information with other species/its role in evolution.

**RE:** We agree with this suggestion. The Discussion has been expanded to better explain the biological significance of A/U-ending codon enrichment and C/G-ending codon suppression. Specifically, we now:

link A/U bias to mutational pressure in RNA viruses.

discuss CpG suppression and immune evasion (e.g., ZAP-mediated targeting).

compare these patterns with observations in other RNA viruses reported in the literature.

Additional references have been incorporated to strengthen this section.

3. Revise the Introduction part thoroughly. Need to add more description related to codon usage.

**RE:** The Introduction has been substantially revised to include a more comprehensive explanation of codon usage bias, including its origins (mutational pressure vs natural selection), its role in viral evolution, and its relevance to host adaptation. Additional references have been incorporated to strengthen the background.

4. The Results and discussion part needs to be thoroughly checked and be uniform in the same

order.

**RE:** We have carefully revised both sections to ensure:

consistent structure and logical flow.

clear separation of Results (descriptive) and Discussion (interpretive)

uniform terminology throughout.

5. The following paper should be discussed.

**RE:** We thank the reviewer for these valuable suggestions. Both studies have now been incorporated into the Discussion section, where we compare our findings with previously reported codon usage patterns in coronaviruses and other RNA viruses.

**Reviewer #2:**

1. Line 13, missing a full stop before “Although”.

**RE:** Corrected

2. Line 56-67. Why can the codon usage analyses of the S genes provide key insights into how

coronaviruses adapt to new hosts? And how did the authors deal with the issue?

**RE:** We have clarified this in the Introduction. The spike (S) protein mediates host receptor binding and entry, making it a key determinant of host specificity and cross-species transmission. To address host adaptation, we used multiple complementary analyses (CAI, ENC, GC3s, RSCU, CA) to distinguish host-associated selection from mutational bias and phylogenetic effects.

3. line 164-165 “Across genera, ENC values ranged from 42.1 to 56.8”, however, when the

reviwer check the S1 Table, we found that the ENC values ranged 32.77 to 55.19. There must be a mistake in this result. I think the figure.6 was plotted based on the S1 Table.

**RE:** We thank the reviewer for identifying this inconsistency. The reported ranges have now been corrected to match the values in Table S3 (revised). All figures and text have been carefully rechecked for consistency.

4. line 173. “In this study, GC3s content varied between 31.8% and 54.6% across all isolates.”

But when we check the results in the S1 Table, they are not consistent.

**RE:** We thank the reviewer for identifying this inconsistency. The reported ranges have now been corrected to match the values in Table S3 (revised). All figures and text have been carefully rechecked for consistency.

5. line 196-197. codon bias (ENC)? ENC is the abbr of “effective number of codons”. The author

needs to clarify the relationship between codon bias and ENC.

**RE:** We have revised the text to clearly state that: ENC is a quantitative measure of codon usage bias. Lower ENC values indicate stronger bias, while higher values indicate weaker bias.

6. line 202, “ENC which is an overall measure of codon usage bias, differs significantly among

genera underscores evolutionary divergence in synonymous codon preference.” , here grammer checking is needed.

**RE:** Corrected.

7. line 249 and 427, all the present manuscript. “host nucleotide composition”, “Host-based

analyses”. how does the CAI calculated in the present study? Samples were derived from different hosts, how to determine which host is the object of the CAI calculation? For instance, could the CAI of Porcine epidemic diarrhea virus_NC_003436.1 calculated based on its adaptation to humans or other animals? And further, are RSCU values of hosts needed if the CAI were calculated?

**RE:** We appreciate this critical point and have clarified the methodology in detail.

CAI was calculated using host-specific codon usage tables as reference sets, based on the most probable natural host of each virus (e.g., *Homo sapiens* for human coronaviruses, *Sus scrofa* for porcine viruses). Importantly, during revision we identified and corrected an error in the reference set used for bat-associated viruses, where an inappropriate dataset had been used. This has now been corrected using the appropriate host reference. Host groups were consolidated into five biologically meaningful categories (bat, avian, human, rodent/insectivore, other mammals) rather than twelve individual species, improving statistical power for post hoc comparisons. All CAI values have been recalculated, and the updated results remain consistent with the overall conclusions of the study. This correction has been explicitly noted in the revised Methods section.

We also clarify that: CAI uses host codon usage tables, not host RSCU values directly. RSCU analysis in this study applies to viral sequences only.

8. line 100. why “Seventy-four (74) reference genomes” changed to the 64 genomes in the Table

S1 and the Figure.4?

**RE:** Clarified in Methods: 74 genomes were initially retrieved, and 64 were retained after quality filtering.

9. Such chaotic results and result analysis require the author to rewrite this paper.

**RE:** We respectfully acknowledge the reviewer’s concern. In response, we have:

thoroughly revised the Results section for clarity and consistency.

corrected all numerical inconsistencies.

ensured alignment between figures, tables, and text.

improved overall structure and readability.

We believe the revised manuscript now presents the findings in a clear, coherent, and logically structured manner.

**Final statement:** We are grateful for the reviewers’ constructive feedback, which has significantly improved the quality of our manuscript. All comments have been carefully addressed, and we believe the revised version is now clearer, more rigorous, and suitable for publication.

**SUMMARY OF REVISIONS**

The revised manuscript incorporates substantive methodological improvements, analytical additions, and editorial corrections across all sections, as detailed below.

**Abstract**

The Abstract has been restructured for greater clarity and conciseness. Specific findings that were previously absent have been added, including: (1) the CpG-containing codon suppression finding from the heatmap analysis, and (2) the ENC–GC3s deviation from the neutral curve, which indicates that factors beyond mutational bias shape codon usage patterns.

**Introduction**

Clarification and expansion of the Introduction, with additional background on codon usage bias, its evolutionary determinants, and relevance to host adaptation.

**Materials and Methods**

Several important methodological details have been added or clarified. (1) The database retrieval date (2nd April 2025) and Biopython version (v1.81) have been specified. (2) Explicit quality control filtering criteria have been added. (3) Host species have been consolidated into five biologically meaningful groups (bat, avian, human, rodent/insectivore, other mammals) to improve statistical power for post hoc comparisons.

**Results**

The Results section has undergone both revisions and substantive additions. (1) The revised results (Figure 1 and 2) consistently report that both CAI and ENC differ significantly among genera (p < 0.05), while GC3s does not, which reflects the updated host-specific CAI analysis. (2) Quantitative codon-level correspondence analysis (CA) interpretation has been added. (3) An explanation for amino acids encoded by only two synonymous codons (Asn, Asp, His, Tyr, Cys, Gln, Phe, Lys) showing no preferred codon in Table 3 has been added, clarifying that near-equal usage in two-codon families does not indicate an error.

**Discussion**

The Discussion has been substantially expanded and restructured to improve its scientific depth and cohesion with the Results. Key additions and corrections include: (1) A direct comparison with Daron & Bravo (2021) has been added to the ENC–GC3s paragraph. While our study confirms their finding of CpG suppression and mutational bias as drivers, our spike-gene-specific analysis reveals translational selection as an additional contributor not identified in whole-genome analyses, likely because gene-specific selective pressures are diluted at the genome level. (2) Biological explanations for the ENC–GC3s outliers have been added with supporting citations: (3) The potential confounding of bat-associated CAI values by the predominance of *Alphacoronaviruses* in that group (19 of 25 bat coronaviruses) has been explicitly acknowledged. (5) The CpG suppression paragraph now cites both Takata et al. (2017) for the ZAP mechanism and Xia (2020) for the coronavirus-specific application.

We believe that the revised manuscript is substantially strengthened by these changes and is now suitable for publication in PLOS ONE. We are grateful for the opportunity to revise and resubmit our work, and we hope that the revisions adequately address all concerns raised.

Yours sincerely,

**Chong Han Ng**

Multimedia University, Malaysia
