# Supplementary material for: Genus- and host-associated codon usage bias patterns in coronavirus spike genes
Source: PLoS One. 2026 Jun 24;21(6):e0351693. doi: 10.1371/journal.pone.0351693 (PMC13293466; doi:10.1371/journal.pone.0351693)
Supplement: Peer review history

# Peer review: Decision Letter 0

**DOI:** 10.1371/journal.pone.0351693.r001

**Authors:** Haitham Mohamed Amer

15 Mar 2026

PONE-D-26-10054

Genus- and host-associated codon usage bias patterns in coronaviruses spike genes

PLOS One

Dear Dr. Ng,

Thank you for submitting your manuscript to PLOS ONE. After careful consideration, we feel that it has merit but does not fully meet PLOS ONE’s publication criteria as it currently stands. Therefore, we invite you to submit a revised version of the manuscript that addresses the points raised during the review process.

Please submit your revised manuscript by  Apr 29 2026 11:59PM. If you will need more time than this to complete your revisions, please reply to this message or contact the journal office at plosone@plos.org. When you're ready to submit your revision, log on to https://www.editorialmanager.com/pone/ and select the 'Submissions Needing Revision' folder to locate your manuscript file.

Please include the following items when submitting your revised manuscript:

- A letter that responds to each point raised by the academic editor and reviewer(s). You should upload this letter as a separate file labeled 'Response to Reviewers'.
- A marked-up copy of your manuscript that highlights changes made to the original version. You should upload this as a separate file labeled 'Revised Manuscript with Track Changes'.
- An unmarked version of your revised paper without tracked changes. You should upload this as a separate file labeled 'Manuscript'.

-->If you would like to make changes to your financial disclosure, please include your updated statement in your cover letter. Guidelines for resubmitting your figure files are available below the reviewer comments at the end of this letter.

If applicable, we recommend that you deposit your laboratory protocols in protocols.io to enhance the reproducibility of your results. Protocols.io assigns your protocol its own identifier (DOI) so that it can be cited independently in the future. For instructions see: https://journals.plos.org/plosone/s/submission-guidelines#loc-laboratory-protocols. Additionally, PLOS ONE offers an option for publishing peer-reviewed Lab Protocol articles, which describe protocols hosted on protocols.io. Read more information on sharing protocols at https://plos.org/protocols?utm_medium=editorial-email&utm_source=authorletters&utm_campaign=protocols.

We look forward to receiving your revised manuscript.

Kind regards,

Haitham Mohamed Amer, PhD

Academic Editor

PLOS One

Journal Requirements:

When submitting your revision, we need you to address these additional requirements.

1. Please ensure that your manuscript meets PLOS ONE's style requirements, including those for file naming. The PLOS ONE style templates can be found at

https://journals.plos.org/plosone/s/file?id=wjVg/PLOSOne_formatting_sample_main_body.pdf and

https://journals.plos.org/plosone/s/file?id=ba62/PLOSOne_formatting_sample_title_authors_affiliations.pdf

2. Please note that PLOS One has specific guidelines on code sharing for submissions in which author-generated code underpins the findings in the manuscript. In these cases, we expect all author-generated code to be made available without restrictions upon publication of the work. Please review our guidelines at https://journals.plos.org/plosone/s/materials-and-software-sharing#loc-sharing-code and ensure that your code is shared in a way that follows best practice and facilitates reproducibility and reuse.

3. Thank you for stating in your Funding Statement:

“This research is supported by Multimedia University, Malaysia, IRFund (grant number MMUI/240116 awarded to Chong Han, Ng). The funder has no role in study design, data analysis, decision to publish or manuscript preparation.”

Please provide an amended statement that declares *all* the funding or sources of support (whether external or internal to your organization) received during this study, as detailed online in our guide for authors at http://journals.plos.org/plosone/s/submit-now. Please also include the statement “There was no additional external funding received for this study.” in your updated Funding Statement.

Please include your amended Funding Statement within your cover letter. We will change the online submission form on your behalf.

4. Thank you for stating the following financial disclosure:

“This research is supported by Multimedia University, Malaysia, IRFund (grant number MMUI/240116 awarded to Chong Han, Ng). The funder has no role in study design, data analysis, decision to publish or manuscript preparation.”

We note that one or more of the authors is affiliated with the funding organization, indicating the funder may have had some role in the design, data collection, analysis or preparation of your manuscript for publication; in other words, the funder played an indirect role through the participation of the co-authors. If the funding organization did not play a role in the study design, data collection and analysis, decision to publish, or preparation of the manuscript and only provided financial support in the form of authors' salaries and/or research materials, please do the following:

a. Review your statements relating to the author contributions, and ensure you have specifically and accurately indicated the role(s) that these authors had in your study. These amendments should be made in the online form.

b. Confirm in your cover letter that you agree with the following statement, and we will change the online submission form on your behalf:

“The funder provided support in the form of salaries for authors [insert relevant initials], but did not have any additional role in the study design, data collection and analysis, decision to publish, or preparation of the manuscript. The specific roles of these authors are articulated in the ‘author contributions’ section.

5. Thank you for stating the following in your Competing Interests section:

“The authors declare that they have no competing interests.”

Please complete your Competing Interests on the online submission form to state any Competing Interests. If you have no competing interests, please state "The authors have declared that no competing interests exist.", as detailed online in our guide for authors at http://journals.plos.org/plosone/s/submit-now

This information should be included in your cover letter; we will change the online submission form on your behalf.

6. Please note that your Data Availability Statement is currently missing the repository name, DOI/accession number of each dataset or a direct link to access each database. If your manuscript is accepted for publication, you will be asked to provide these details on a very short timeline. We therefore suggest that you provide this information now, though we will not hold up the peer review process if you are unable.

7. If the reviewer comments include a recommendation to cite specific previously published works, please review and evaluate these publications to determine whether they are relevant and should be cited. There is no requirement to cite these works unless the editor has indicated otherwise.

8. Please review your reference list to ensure that it is complete and correct. If you have cited papers that have been retracted, please include the rationale for doing so in the manuscript text, or remove these references and replace them with relevant current references. Any changes to the reference list should be mentioned in the rebuttal letter that accompanies your revised manuscript. If you need to cite a retracted article, indicate the article’s retracted status in the References list and also include a citation and full reference for the retraction notice.

Reviewers' comments:

Reviewer's Responses to Questions

**Comments to the Author**

1. Is the manuscript technically sound, and do the data support the conclusions?

The manuscript must describe a technically sound piece of scientific research with data that supports the conclusions. Experiments must have been conducted rigorously, with appropriate controls, replication, and sample sizes. The conclusions must be drawn appropriately based on the data presented.

Reviewer #1: Yes

Reviewer #2: Partly

**********

2. Has the statistical analysis been performed appropriately and rigorously?

Reviewer #1: Yes

Reviewer #2: No

**********

3. Have the authors made all data underlying the findings in their manuscript fully available?

The PLOS Data policy requires authors to make all data underlying the findings described in their manuscript fully available without restriction, with rare exception (please refer to the Data Availability Statement in the manuscript PDF file). The data should be provided as part of the manuscript or its supporting information, or deposited to a public repository. For example, in addition to summary statistics, the data points behind means, medians and variance measures should be available. If there are restrictions on publicly sharing data—e.g. participant privacy or use of data from a third party—those must be specified.

Reviewer #1: Yes

Reviewer #2: No

**********

4. Is the manuscript presented in an intelligible fashion and written in standard English?

PLOS ONE does not copyedit accepted manuscripts, so the language in submitted articles must be clear, correct, and unambiguous. Any typographical or grammatical errors should be corrected at revision, so please note any specific errors here.

Reviewer #1: Yes

Reviewer #2: Yes

**********

5. Review Comments to the Author

Please use the space provided to explain your answers to the questions above. You may also include additional comments for the author, including concerns about dual publication, research ethics, or publication ethics. (Please upload your review as an attachment if it exceeds 20,000 characters)

Reviewer #1: I have reviewed the manuscript ID PONE-D-26-10054 titled "Genus- and host-associated codon usage bias patterns in coronaviruses spike genes" submitted by Jia Jun Chew and collaborator. The paper describes codon usage bias patterns in coronaviruses spike genes associated to its hosts. Codon usage patterns were assessed of Alphacoronavirus, Betacoronavirus, Gammacoronavirus, and Deltacoronavirus using effective number of codons (ENC), relative synonymous codon usage (RSCU), codon adaptation index (CAI), correspondence analysis, hierarchical clustering, and GC content at synonymous third codon positions (GC3s) modelling. The authors pointing out the dual role of mutation and natural selection in coronavirus evolution, with host-driven selection as a major determinant of codon bias.

Although the work described has significance, the manuscript is well written. However, in some area needs to be polished as there are grammatical errors in it.

What was reason behind confining study for 74 reference genome?

Highlight the significance of synonymous codons ending in A/T & C/G in the discussion and provide similarity information with other species/its role in evolution.

Revise the Introduction part thoroughly. Need to add more description related to codon usage.

The Results and discussion part needs to be thoroughly checked and be uniform in the same order.

The following paper should be discussed.

doi: 10.3389/fvets.2022.1021440

https://doi.org/10.1016/j.meegid.2017.11.027

Reviewer #2: 1. Line 13, missing a full stop before “Although”.

2. Line 56-67. Why can the codon usage analyses of the S genes provide key insights into how coronaviruses adapt to new hosts? And how did the authors deal with the issue?

3. line 164-165 “Across genera, ENC values ranged from42.1 to 56.8”,however, when the reviwer check the S1 Table, we found that the ENC values ranged 32.77 to 55.19. There must be a mistake in this result. I think the figure.6 was plotted based on the S1 Table.

4. line 173. “In this study, GC3s content varied between 31.8% and 54.6% across all isolates.” But when we check the results in the S1 Table, they are not consistent.

5. line 196-197. codon bias (ENC)? ENC is the abbr of “effective number of codons”. The author needs to clarify the relationship between codon bias and ENC.

6. line 202, “ENC which is an overall measure of codon usage bias, differs significantly among genera underscores evolutionary divergence in synonymous codon preference.” , here grammer checking is needed.

7. line 249 and 427, all the present manuscript. “host nucleotide composition”, “Host-based analyses”. how does the CAI calculated in the present study? Samples were derived from different hosts, how to determine which host is the object of the CAI calculation? For instance, could the CAI of Porcine epidemic diarrhea virus_NC_003436.1 calculated based on its adaptation to humans or other animals? And further, are RSCU values of hosts needed if the CAI were calculated?

8. line 100. why “Seventy-four (74) reference genomes” changed to the 64 genomes in the Table S1 and the Figure.4?

9. Such chaotic results and result analysis require the author to rewrite this paper.

**********

6. PLOS authors have the option to publish the peer review history of their article (what does this mean?). If published, this will include your full peer review and any attached files.

If you choose “no”, your identity will remain anonymous but your review may still be made public.

**Do you want your identity to be public for this peer review?**  For information about this choice, including consent withdrawal, please see our Privacy Policy.

Reviewer #1: No

Reviewer #2: No

**********

[NOTE: If reviewer comments were submitted as an attachment file, they will be attached to this email and accessible via the submission site. Please log into your account, locate the manuscript record, and check for the action link "View Attachments". If this link does not appear, there are no attachment files.]

To ensure your figures meet our technical requirements, please review our figure guidelines: https://journals.plos.org/plosone/s/figures

You may also use PLOS’s free figure tool, NAAS, to help you prepare publication quality figures: https://journals.plos.org/plosone/s/figures#loc-tools-for-figure-preparation.

NAAS will assess whether your figures meet our technical requirements by comparing each figure against our figure specifications.

# Author comment: Author response to Decision Letter 1

**DOI:** 10.1371/journal.pone.0351693.r002

16 May 2026

RESPONSES TO REVIEWERS COMMENTS

We sincerely thank the reviewers for their careful evaluation and constructive comments. We have revised the manuscript accordingly and believe that these changes have significantly improved its clarity, accuracy, and overall quality. All changes have been incorporated into the revised manuscript and are highlighted where appropriate.

Reviewer #1: I have reviewed the manuscript ID PONE-D-26-10054 titled "Genus- and host-associated codon usage bias patterns in coronaviruses spike genes" submitted by Jia Jun Chew and collaborator. The paper describes codon usage bias patterns in coronaviruses spike genes associated to its hosts. Codon usage patterns were assessed of Alphacoronavirus, Betacoronavirus, Gammacoronavirus, and Deltacoronavirus using effective number of codons (ENC), relative synonymous codon usage (RSCU), codon adaptation index (CAI), correspondence analysis, hierarchical clustering, and GC content at synonymous third codon positions (GC3s) modelling. The authors pointing out the dual role of mutation and natural selection in coronavirus evolution, with host-driven selection as a major determinant of codon bias.

Although the work described has significance, the manuscript is well written. However, in some area needs to be polished as there are grammatical errors in it.

1. What was reason behind confining study for 74 reference genome?

RE: We thank the reviewer for this important question. The dataset was not arbitrarily limited to 74 genomes. Instead, all available reference genomes under the query “Orthocoronavirinae” were retrieved from the NCBI Virus database to ensure high-quality, well-annotated, and non-redundant sequences across the four coronavirus genera. Orthocoronavirinae is the currently accepted subfamily name in modern coronavirus taxonomy. “Coronavirinae” is an older classification that was revised by the International Committee on Taxonomy of Viruses.

To avoid overrepresentation of closely related strains, one representative genome per species was selected. After quality filtering (removal of incomplete or low-quality spike CDS), 64 genomes were retained for downstream analyses. This clarification has now been added to the Materials and Methods section.

2. Highlight the significance of synonymous codons ending in A/T & C/G in the discussion and

provide similarity information with other species/its role in evolution.

RE: We agree with this suggestion. The Discussion has been expanded to better explain the biological significance of A/U-ending codon enrichment and C/G-ending codon suppression. Specifically, we now:

link A/U bias to mutational pressure in RNA viruses.

discuss CpG suppression and immune evasion (e.g., ZAP-mediated targeting).

compare these patterns with observations in other RNA viruses reported in the literature.

Additional references have been incorporated to strengthen this section.

3. Revise the Introduction part thoroughly. Need to add more description related to codon usage.

RE: The Introduction has been substantially revised to include a more comprehensive explanation of codon usage bias, including its origins (mutational pressure vs natural selection), its role in viral evolution, and its relevance to host adaptation. Additional references have been incorporated to strengthen the background.

4. The Results and discussion part needs to be thoroughly checked and be uniform in the same

order.

RE: We have carefully revised both sections to ensure:

consistent structure and logical flow.

clear separation of Results (descriptive) and Discussion (interpretive)

uniform terminology throughout.

5. The following paper should be discussed.

RE: We thank the reviewer for these valuable suggestions. Both studies have now been incorporated into the Discussion section, where we compare our findings with previously reported codon usage patterns in coronaviruses and other RNA viruses.

Reviewer #2:

1. Line 13, missing a full stop before “Although”.

RE: Corrected

2. Line 56-67. Why can the codon usage analyses of the S genes provide key insights into how

coronaviruses adapt to new hosts? And how did the authors deal with the issue?

RE: We have clarified this in the Introduction. The spike (S) protein mediates host receptor binding and entry, making it a key determinant of host specificity and cross-species transmission. To address host adaptation, we used multiple complementary analyses (CAI, ENC, GC3s, RSCU, CA) to distinguish host-associated selection from mutational bias and phylogenetic effects.

3. line 164-165 “Across genera, ENC values ranged from 42.1 to 56.8”, however, when the

reviwer check the S1 Table, we found that the ENC values ranged 32.77 to 55.19. There must be a mistake in this result. I think the figure.6 was plotted based on the S1 Table.

RE: We thank the reviewer for identifying this inconsistency. The reported ranges have now been corrected to match the values in Table S3 (revised). All figures and text have been carefully rechecked for consistency.

4. line 173. “In this study, GC3s content varied between 31.8% and 54.6% across all isolates.”

But when we check the results in the S1 Table, they are not consistent.

RE: We thank the reviewer for identifying this inconsistency. The reported ranges have now been corrected to match the values in Table S3 (revised). All figures and text have been carefully rechecked for consistency.

5. line 196-197. codon bias (ENC)? ENC is the abbr of “effective number of codons”. The author

needs to clarify the relationship between codon bias and ENC.

RE: We have revised the text to clearly state that: ENC is a quantitative measure of codon usage bias. Lower ENC values indicate stronger bias, while higher values indicate weaker bias.

6. line 202, “ENC which is an overall measure of codon usage bias, differs significantly among

genera underscores evolutionary divergence in synonymous codon preference.” , here grammer checking is needed.

RE: Corrected.

7. line 249 and 427, all the present manuscript. “host nucleotide composition”, “Host-based

analyses”. how does the CAI calculated in the present study? Samples were derived from different hosts, how to determine which host is the object of the CAI calculation? For instance, could the CAI of Porcine epidemic diarrhea virus_NC_003436.1 calculated based on its adaptation to humans or other animals? And further, are RSCU values of hosts needed if the CAI were calculated?

RE: We appreciate this critical point and have clarified the methodology in detail.

CAI was calculated using host-specific codon usage tables as reference sets, based on the most probable natural host of each virus (e.g., Homo sapiens for human coronaviruses, Sus scrofa for porcine viruses). Importantly, during revision we identified and corrected an error in the reference set used for bat-associated viruses, where an inappropriate dataset had been used. This has now been corrected using the appropriate host reference. Host groups were consolidated into five biologically meaningful categories (bat, avian, human, rodent/insectivore, other mammals) rather than twelve individual species, improving statistical power for post hoc comparisons. All CAI values have been recalculated, and the updated results remain consistent with the overall conclusions of the study. This correction has been explicitly noted in the revised Methods section.

We also clarify that: CAI uses host codon usage tables, not host RSCU values directly. RSCU analysis in this study applies to viral sequences only.

8. line 100. why “Seventy-four (74) reference genomes” changed to the 64 genomes in the Table

S1 and the Figure.4?

RE: Clarified in Methods: 74 genomes were initially retrieved, and 64 were retained after quality filtering.

9. Such chaotic results and result analysis require the author to rewrite this paper.

RE: We respectfully acknowledge the reviewer’s concern. In response, we have:

thoroughly revised the Results section for clarity and consistency.

corrected all numerical inconsistencies.

ensured alignment between figures, tables, and text.

improved overall structure and readability.

We believe the revised manuscript now presents the findings in a clear, coherent, and logically structured manner.

Final statement: We are grateful for the reviewers’ constructive feedback, which has significantly improved the quality of our manuscript. All comments have been carefully addressed, and we believe the revised version is now clearer, more rigorous, and suitable for publication.

SUMMARY OF REVISIONS

The revised manuscript incorporates substantive methodological improvements, analytical additions, and editorial corrections across all sections, as detailed below.

Abstract

The Abstract has been restructured for greater clarity and conciseness. Specific findings that were previously absent have been added, including: (1) the CpG-containing codon suppression finding from the heatmap analysis, and (2) the ENC–GC3s deviation from the neutral curve, which indicates that factors beyond mutational bias shape codon usage patterns.

Introduction

Clarification and expansion of the Introduction, with additional background on codon usage bias, its evolutionary determinants, and relevance to host adaptation.

Materials and Methods

Several important methodological details have been added or clarified. (1) The database retrieval date (2nd April 2025) and Biopython version (v1.81) have been specified. (2) Explicit quality control filtering criteria have been added. (3) Host species have been consolidated into five biologically meaningful groups (bat, avian, human, rodent/insectivore, other mammals) to improve statistical power for post hoc comparisons.

Results

The Results section has undergone both revisions and substantive additions. (1) The revised results (Figure 1 and 2) consistently report that both CAI and ENC differ significantly among genera (p < 0.05), while GC3s does not, which reflects the updated host-specific CAI analysis. (2) Quantitative codon-level correspondence analysis (CA) interpretation has been added. (3) An explanation for amino acids encoded by only two synonymous codons (Asn, Asp, His, Tyr, Cys, Gln, Phe, Lys) showing no preferred codon in Table 3 has been added, clarifying that near-equal usage in two-codon families does not indicate an error.

Discussion

The Discussion has been substantially expanded and restructured to improve its scientific depth and cohesion with the Results. Key additions and corrections include: (1) A direct comparison with Daron & Bravo (2021) has been added to the ENC–GC3s paragraph. While our study confirms their finding of CpG suppression and mutational bias as drivers, our spike-gene-specific analysis reveals translational selection as an additional contributor not identified in whole-genome analyses, likely because gene-specific selective pressures are diluted at the genome level. (2) Biological explanations for the ENC–GC3s outliers have been added with supporting citations: (3) The potential confounding of bat-associated CAI values by the predominance of Alphacoronaviruses in that group (19 of 25 bat coronaviruses) has been explicitly acknowledged. (5) The CpG suppression paragraph now cites both Takata et al. (2017) for the ZAP mechanism and Xia (2020) for the coronavirus-specific application.

We believe that the revised manuscript is substantially strengthened by these changes and is now suitable for publication in PLOS ONE. We are grateful for the opportunity to revise and resubmit our work, and we hope that the revisions adequately address all concerns raised.

Yours sincerely,

Chong Han Ng

Multimedia University, Malaysia

Attachment Submitted filename: Response to Reviewers.docx

# Peer review: Decision Letter 1

**DOI:** 10.1371/journal.pone.0351693.r003

**Authors:** Haitham Mohamed Amer

31 May 2026

<p>Genus- and host-associated codon usage bias patterns in coronavirus spike genes

PONE-D-26-10054R1

Dear Dr. Ng,

We’re pleased to inform you that your manuscript has been judged scientifically suitable for publication and will be formally accepted for publication once it meets all outstanding technical requirements.

Within one week, you’ll receive an e-mail detailing the required amendments. When these have been addressed, you’ll receive a formal acceptance letter and your manuscript will be scheduled for publication.

An invoice will be generated when your article is formally accepted. Please note, if your institution has a publishing partnership with PLOS and your article meets the relevant criteria, all or part of your publication costs will be covered. Please make sure your user information is up-to-date by logging into Editorial Manager at Editorial Manager® and clicking the ‘Update My Information' link at the top of the page. For questions related to billing, please contact billing support.

If your institution or institutions have a press office, please notify them about your upcoming paper to help maximize its impact. If they’ll be preparing press materials, please inform our press team as soon as possible -- no later than 48 hours after receiving the formal acceptance. Your manuscript will remain under strict press embargo until 2 pm Eastern Time on the date of publication. For more information, please contact onepress@plos.org.

Kind regards,

Haitham Mohamed Amer, PhD

Academic Editor

PLOS One

# Editor report: Acceptance letter

**DOI:** 10.1371/journal.pone.0351693.r004

**Authors:** Haitham Mohamed Amer

PONE-D-26-10054R1

PLOS One

Dear Dr. Ng,

I'm pleased to inform you that your manuscript has been deemed suitable for publication in PLOS One. Congratulations! Your manuscript is now being handed over to our production team.

At this stage, our production department will prepare your paper for publication. This includes ensuring the following:

* All references, tables, and figures are properly cited

* All relevant supporting information is included in the manuscript submission,

* There are no issues that prevent the paper from being properly typeset

You will receive further instructions from the production team, including instructions on how to review your proof when it is ready. Please keep in mind that we are working through a large volume of accepted articles, so please give us a few days to review your paper and let you know the next and final steps.

Lastly, if your institution or institutions have a press office, please let them know about your upcoming paper now to help maximize its impact. If they'll be preparing press materials, please inform our press team within the next 48 hours. Your manuscript will remain under strict press embargo until 2 pm Eastern Time on the date of publication. For more information, please contact onepress@plos.org.

You will receive an invoice from PLOS for your publication fee after your manuscript has reached the completed accept phase. If you receive an email requesting payment before acceptance or for any other service, this may be a phishing scheme. Learn how to identify phishing emails and protect your accounts at https://explore.plos.org/phishing.

If we can help with anything else, please email us at customercare@plos.org.

Thank you for submitting your work to PLOS ONE and supporting open access.

Kind regards,

PLOS ONE Editorial Office Staff

on behalf of

Dr. Haitham Mohamed Amer

Academic Editor

PLOS One
